# Efficacy of Intravenous Ustekinumab Reinduction in Patients With Crohn’s Disease With a Loss of Response

**DOI:** 10.1093/jcag/gwac017

**Published:** 2022-05-26

**Authors:** Valerie Heron, Steven Li Fraine, Nicola Panaccione, Sophie Restellini, Pascale Germain, Kristina Candido, Charles N Bernstein, Talat Bessissow, Alain Bitton, Usha K Chauhan, Peter L Lakatos, John K Marshall, Pierre Michetti, Cynthia H Seow, Greg Rosenfeld, Remo Panaccione, Waqqas Afif

**Affiliations:** Division of Gastroenterology, Hôpital Maisonneuve-Rosemont, Université de Montréal, Montreal, Quebec, Canada; Inflammatory Bowel Disease Centre, Division of Gastroenterology, McGill University Health Centre (MUHC), Montreal, Quebec, Canada; Inflammatory Bowel Disease Centre, Division of Gastroenterology, McGill University Health Centre (MUHC), Montreal, Quebec, Canada; Inflammatory Bowel Disease Unit, Division of Gastroenterology and Hepatology, University of Calgary, Calgary, AlbertaCanada; Division of Gastroenterology and Hepatology, Geneva’s University Hospitals and University of Geneva, Switzerland; Inflammatory Bowel Disease Centre, Division of Gastroenterology, McGill University Health Centre (MUHC), Montreal, Quebec, Canada; Inflammatory Bowel Disease Centre, Division of Gastroenterology, McGill University Health Centre (MUHC), Montreal, Quebec, Canada; Department of Medicine, Section of Gastroenterology, Max Rady College of Medicine, Rad Faculty of Health Sciences, University of Manitoba, and the University of Manitoba IBD Clinical and Research Centre, Winnipeg, Manitoba, Canada; Inflammatory Bowel Disease Centre, Division of Gastroenterology, McGill University Health Centre (MUHC), Montreal, Quebec, Canada; Inflammatory Bowel Disease Centre, Division of Gastroenterology, McGill University Health Centre (MUHC), Montreal, Quebec, Canada; Department of Medicine (Division of Gastroenterology) and Farncombe Family Digestive Health Research Institute, McMaster University, Hamilton, Ontario, Canada; Inflammatory Bowel Disease Centre, Division of Gastroenterology, McGill University Health Centre (MUHC), Montreal, Quebec, Canada; Department of Medicine (Division of Gastroenterology) and Farncombe Family Digestive Health Research Institute, McMaster University, Hamilton, Ontario, Canada; Gastroenterology Beaulieu and Division of Gastroenterology and Hepatology, CHUV, Lausanne, Switzerland; Inflammatory Bowel Disease Unit, Division of Gastroenterology and Hepatology, University of Calgary, Calgary, AlbertaCanada; Division of Gastroenterology, University of British Columbia, Vancouver, British Columbia, Canada; Inflammatory Bowel Disease Unit, Division of Gastroenterology and Hepatology, University of Calgary, Calgary, AlbertaCanada; Inflammatory Bowel Disease Centre, Division of Gastroenterology, McGill University Health Centre (MUHC), Montreal, Quebec, Canada

**Keywords:** Crohn’s disease, Reinduction, Therapeutic drug monitoring, Ustekinumab

## Abstract

**Background/Aims:**

In patients receiving ustekinumab (UST) for treatment of Crohn’s disease, there is no proven strategy to enhance or re-capture response. We assessed the utility of UST intravenous (IV) reinduction (~6 mg/kg) to achieve clinical, biochemical and endoscopic response or remission, in patients with partial or loss of response to UST maintenance therapy.

**Methods:**

A multicentre, retrospective cohort study was performed. Adults who received an IV reinduction dose of UST for either partial response or secondary loss of response to UST were assessed. The primary outcome was clinical remission off corticosteroids (Harvey Bradshaw Index <5), with biochemical response (defined as ≥ 50% decrease of CRP or FCP and/or endoscopic response (defined as a decrease in Simple Endoscopic Score-CD ≥ 50%). Secondary outcomes included clinical, biomarker and endoscopic response/remission, as well as safety.

**Results:**

Sixty-five patients (median age 38 years, 54.7% women) underwent IV UST reinduction between January 2017 and April 2019. Most patients (88.3%) were already on escalated maintenance dosing of UST 90 mg subcutaneous every 4 weeks. Clinical outcomes were assessed at a median of 14 weeks (IQR: 12–19) post-reinduction. The primary outcome of clinical remission off corticosteroids with biochemical and/or endoscopic response was achieved in 31.0% (*n* = 18). Pre-reinduction UST concentrations were ≥1 μg/mL in 88.6% (mean 3.2 ± 2.0 μg/mL). No serious adverse events were reported.

**Conclusions:**

UST IV reinduction can be effective in patients with Crohn’s disease with partial or loss of response to UST maintenance therapy. Further studies evaluating this strategy are warranted.

## INTRODUCTION

Crohn’s disease is an immune-mediated condition characterized by chronic bowel inflammation (1,2). Several biologic therapies aim to modulate various inflammatory pathways involved in the disease process and are approved for inducing and maintaining remission of Crohn’s disease (3). However, secondary loss of response (LOR) remains a significant problem. For anti-TNF therapy, the annual rate of LOR is 10%–20% per year (4,5). Therapeutic drug monitoring with resultant dose intensification, addition of an immunomodulator or switching to another biologic have been used to recapture response and remission. Dose intensification with anti-TNF may be achieved by increasing the dose or decreasing the dose interval with short term response rates of up to 70% (6). There are fewer data on reinduction with anti-TNF medications, but short-term dose escalation can be equally effective and less costly than sustained dose intensification (7).

Ustekinumab (UST) is a monoclonal antibody that inhibits the p40 subunit common to interleukin (IL)-12 and IL-23 (8). UST has been demonstrated to safely and effectively induce and maintain remission in patients with Crohn’s disease (CD) in clinical trials (9). The risk of secondary LOR to UST has not been extensively investigated. In the long-term extension study of IM-UNITI, patients on UST q 8 week therapy maintained clinical remission from weeks 44 to 92 (84.1% to 74.4%) (10). Sustained clinical remission at three years was found in 55.1% of randomised and non-randomised patients treated with UST q 8 weeks (11). Results from pooled real-world data have shown a clinical response rate of 49% (95% CI: 0.37–0.62) at 52 weeks and an endoscopic response rate of 63% (95% CI: 0.53–0.72) after approximately 1 year in a population of predominantly anti-TNF-experienced patients (97.7%) (12). In a more recent study, short-term clinical response and remission after dose escalation to every 4 weeks were seen in 61% and 31% of patients (13).

In clinical practice, patients with CD may experience an incomplete response or lose response to UST over time. We assessed the efficacy of intravenous (IV) UST reinduction to achieve clinical and endoscopic response or remission in patients with active CD on UST maintenance therapy.

## METHODS

### Study Design and Setting

Adult patients receiving UST for the treatment of Crohn’s disease who received an IV reinduction of UST (standard weight-based dosing) for sub-optimal response or a loss of response at the discretion of their physician were identified from five sites of the Canadian IBD Research Consortium (CIRC) network (Montreal, Hamilton, Winnipeg, Calgary and Vancouver) and from Switzerland (Geneva). Intravenous reinduction dosing was provided by Janssen as compassionate use, but there was no additional industry support for this study. Inclusion criteria included any patient who received IV UST reinduction and had at least one follow-up with at least one of the outcomes assessed.

### Variables

Demographic data including age, gender, smoking status, disease characteristics (Montreal classification), prior surgeries, and prior medical therapies were collected via retrospective chart review at each site. Concomitant therapies at the time of reinduction were recorded. Disease activity at the time of reinduction was assessed clinically using the Harvey Bradshaw Index (HBI) calculated based on retrospective chart review. If the HBI was unable to be calculated, clinical activity was based off the treating physician’s evaluation. Serum C-reactive protein (CRP) and fecal calprotectin (FCP) values, as well as available endoscopic assessments were recorded. When available, serum UST levels and anti-UST antibody titres were also collected. An adequate UST level was defined as ≥1 μg/mL, as suggested by exposure-response analyses of UNITI subjects (9,14).

### Outcomes

The primary outcome of interest was the composite outcome of clinical remission with either biochemical and/or endoscopic response or remission, following UST IV reinduction. Clinical remission was defined as an HBI <5 with no use of corticosteroids at the time of evaluation. Biochemical remission was defined as normalization of available biomarkers, FCP (<250 μg/g) and/or CRP (normal defined by local lab cut-offs). Endoscopic remission was defined as a simple endoscopic score for CD (SES-CD) <3. Clinical, biochemical and endoscopic response were defined as ≥50% decreases in HBI, biomarker levels and SES-CD score, respectively. Secondary outcomes included clinical, biomarker, and endoscopic response and remission, as well as safety assessments.

### Statistical Analysis

Research Ethics Board approval was obtained at each participating site and data transfer agreements were obtained for confidential communication of patient information between sites. Data collected at each site were aggregated and analyzed collectively. Drug discontinuation was considered as a failure to achieve the primary outcome. Only patients with available pre- and post-induction data were included in the analysis of individual outcomes. Predictors of remission were assessed and included age, sex, weight, smoking status, type of inductions (IV or SC), concurrent use of immunomodulators, HBI pre-induction, CRP pre-induction, and pre-induction UST concentration. Statistical analysis was performed using SPSS statistical software package version 24.0 (IBM, New York NY). Descriptive analysis (median and interquartile range [IQR] for continuous data or percentage for dichotomous) were performed. Continuous variables were analyzed by a 2-tailed t test. All *P* values were 2-tailed, and *P* value <0.05 were considered statistically significant.

## RESULTS

### Baseline Characteristics

A total of 65 patients (median age 38 years, 54.7% women) underwent IV UST reinduction between January 2017 and April 2019. Baseline demographic data are listed in Table 1. All patients included had previously failed at least one anti-TNF therapy. Most patients (88.3%, *n* = 53) were already on escalated maintenance dosing of UST 90 mg subcutaneous (SC) every 4 weeks at the time that they received their IV reinduction. Nearly half of the patients (46.2%, *n* = 30) had originally received an IV induction at initiation of UST therapy while the rest had received a high dose subcutaneous induction regimen prior to IV UST becoming available. The indication for reinduction (when available) was reported to be partial response in 40.0% (*n* = 30) and LOR in 47.7% (*n* = 35). Post-reinduction, 84.6% (*n* = 55) were maintained on q 4-week dosing, 1.5% (*n* = 1) on q 6- week dosing, and 13.8% (*n* = 9) on q 8-week dosing. The median time from initiating UST to receiving the IV reinduction was 15 months (interquartile range [IQR]: 9–29). HBI post reinduction scores were available in 78.5% (*n* = 51) of patients. Clinical outcomes were assessed at a median of 14 weeks (IQR: 12-19) post-reinduction.

**Table 1.** Baseline demographic data

		Available data (total n, missing n)
**Median age at reinduction** (y, IQR)	38.0 (24-44)	64 (1)
**Female gender** (n, %)	35, 54.7%	64 (1)
**Smoking status**		58 (7)
Current (n, %)	5,9.0%	
Former (n, %)	9, 16%	
**Montreal classification**		
**Age at diagnosis**		62 (3)
≤16 years (n, %)	19, 30.6%	
17-40 years (n, %)	39, 62.9%	
>40 years (n, %)	4, 6.5%	
**Disease location**		
Ileal (n, %)	10, 16.1%	62 (3)
Colonic (n, %)	17, 27.4%	
Ileocolonic (n, %)	35, 56.5%	
Upper GI (n, %)	1, 1.6%	
**Disease behaviour**		62 (3)
Non-stricturing, non-penetrating (n, %)	25, 40.3%	
Stricturing (n, %)	25, 40.3%	
Penetrating (n, %)	12, 19.3%	
Perianal disease (n, %)	24, 38.7%	
**Baseline indices**		
Median HBI pre-reinduction (y, IQR)	8 (5-10)	51 (14)
Median CRP level (mg/L) (y, IQR)	9.5 (3.8-16.2)	63 (2)
Median calprotectin level (mcg/g) (y, IQR)	600 (272-1173)	45 (20)
Median SES-CD (y, IQR)	9 (7-14)	40 (20)
**Prior therapy**		
Prior anti-TNF therapy (n, %)	65, 100%	65 (0)
Failed 2 biologics	48, 74%	65 (0)
Failed 3 or more biologics (n, %)	9, 13.8%	65 (0)
Previous IBD-related surgery (n, %)	30, 46.9%	64 (1)
Ileostomy (n, %)	4, 6.0%	64 (1)
**Corticosteroid use at the time of reinduction**		65 (0)
Prednisone (n, %)	13, 20%	
Budesonide (n, %)	7, 10.8%	
**Immunosuppressant use at the time of reinduction** (n, %)	22, 33.8%	65 (0)
**Type of initial UST induction**		
**Subcutaneous**	34, 53.1%	64 (1)
**Intravenous**	30, 46.9%	
**UST maintenance dosing of q 4 weeks prior to reinduction**(n, %)	53, 88.3%	64 (1)
**Median time from initial induction to IV reinduction** (months, IQR)	15 (9-29)	65 (0)

### Response and Remission Rates Following IV Reinduction

**Figure 1.**
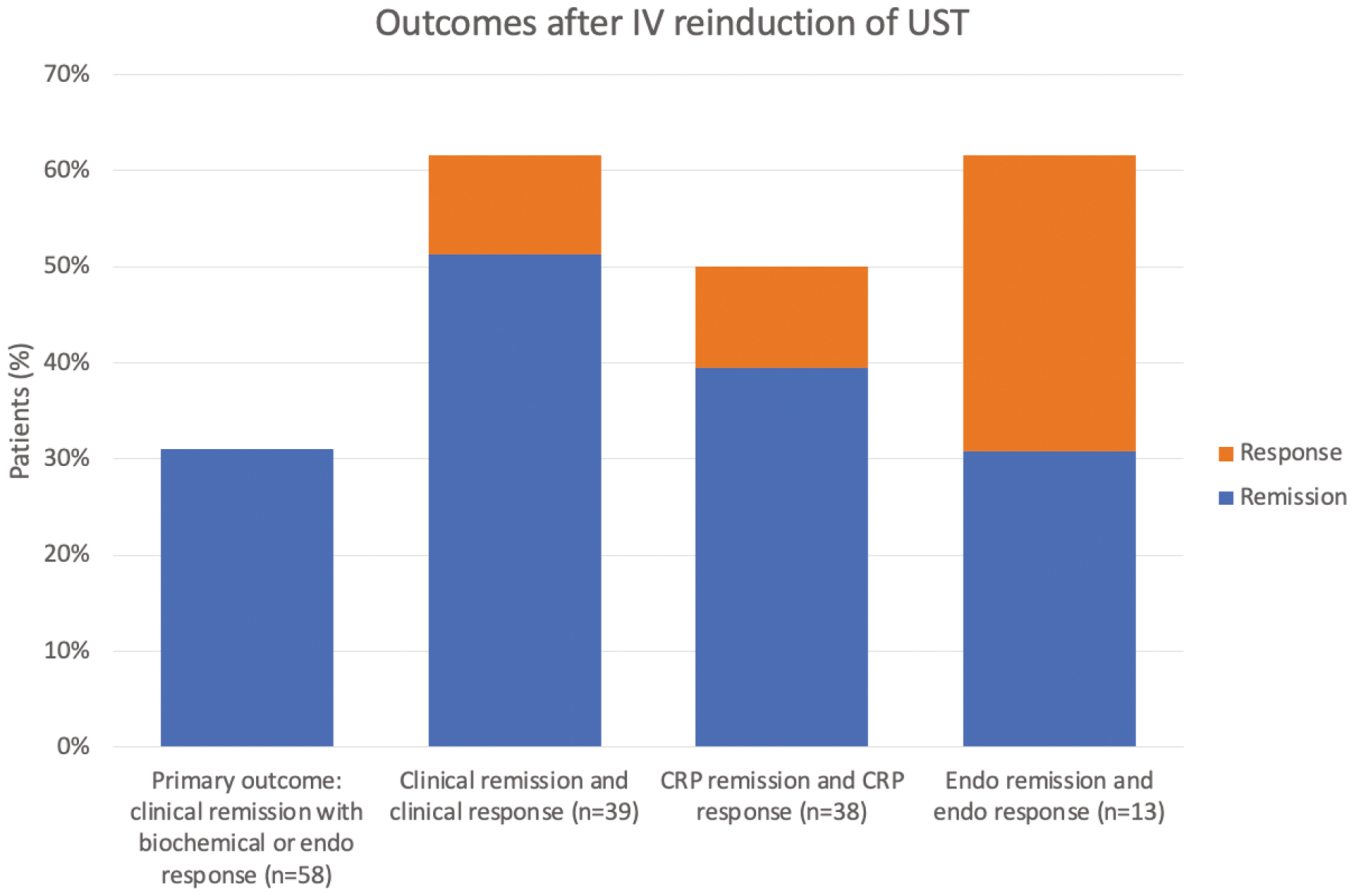
Outcomes post IV re-induction. 224 × 291 mm (96 × 96 DPI).

At the time of follow-up, when assessment of response was performed, 58 of 65 patients (89%) remained on UST maintenance therapy. The seven patients who discontinued UST were considered treatment failures. The primary outcome of clinical remission with either biochemical and endoscopic response or remission at the time of assessment was achieved in 31.0% (*n* = 18) of 58 patients with available data. The primary outcome and individual outcomes after IV UST reinduction of UST are shown in Figure 1. The primary outcome was achieved to a similar degree in both patients who initially received an initial IV induction (27.6%, *n* = 8) or high-dose SC induction (35.7%, n=10).

Among patients who had a recorded HBI ≥5 at baseline and documented HBI at follow-up (*n* = 39), clinical remission was achieved in 51.3% (*n* = 20) and an additional 10.3% (*n* = 4) achieved response. In patients with an elevated CRP documented at baseline and a post-reinduction CRP for comparison (*n* = 38); 50% (*n* = 19) had normalization (39.5%, *n* = 15) or a decrease of at least 50% of their CRP (10.5%, *n* = 4) following reinduction. When FCP was high at baseline with follow-up FCP available (*n* = 24), rates of normal FCP post-reinduction were 33.3% and an additional 16.7% of patients demonstrated a decrease in FCP of ≥50%. When endoscopic data were available pre- and post-reinduction, patients with a high SES-CD score at baseline (*n* = 13) had a 30.8% rate of endoscopic remission and a 30.8% rate of endoscopic response at follow-up. The combined outcome of clinical remission and CRP response or remission in patients with high HBI and/or CRP at baseline (*n* = 53) was achieved in 43.4% of patients. The combined outcome of clinical remission and response/remission of all biomarkers was achieved in 35.7% of patients (*n* = 20). One of 10 patients with active perianal disease at baseline was noted to have improvement of perianal fistulas. Of the 16 patients that were initially on corticosteroids (prednisone or budesonide) and remained on UST maintenance therapy, 12.5% (*n* = 2) still required corticosteroids at follow-up and were assessed as not achieving the primary endpoint.

A sub-analysis looking at the response of UST reinduction according to disease behaviour was performed. The primary outcome was achieved in 42.3% (*n* = 11) of 26 patients with stricturing disease compared to 16.7% (*n* = 2) of 12 patients with penetrating disease and 22.7% (*n* = 5) of 22 patients with non-stricturing, non-penetrating disease. None of the three patients with both stricturing and penetrating disease achieved the primary endpoint. In patients with perianal disease, the primary outcome was reached in 30.4% (*n* = 7) of 23 patients compared to 31.4% (*n* = 11) of 35 patients without perianal involvement.

**Figure 2.**
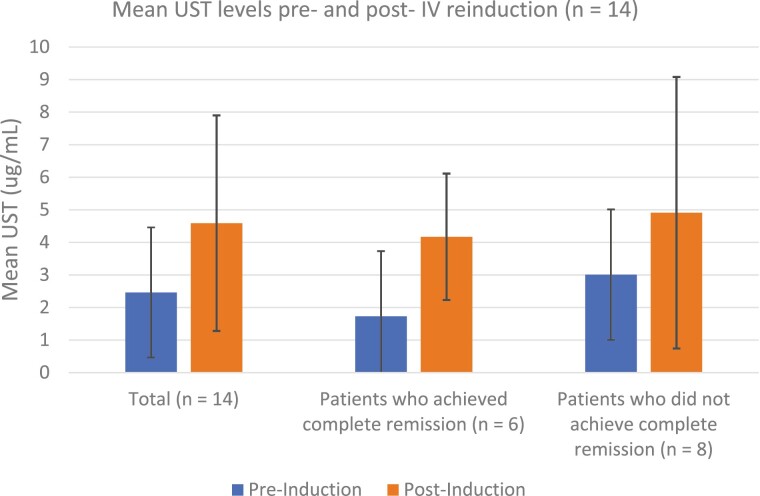
Mean UST concentration pre- and post-IV reinduction. 224 × 291 mm (96 × 96 DPI).

Therapeutic drug monitoring (TDM) for UST was performed in 35 patients prior to reinduction, and 25 patients post-reinduction. The median time of measurement post-reinduction was 8.4 weeks (IQR 4.6–13.5 weeks). Pre-reinduction UST concentrations were ≥1 μg/mL in 88.6% (*n* = 31; mean 3.2 ± 2.0 μg/mL), compared to 96% (*n* = 24) of post-reinduction UST concentrations (mean 4.6 ± 2.7 µg/mL). In the 14 patients where both pre- and post-reinduction UST concentrations were available, the mean pre-induction concentrations in patients achieving the primary outcome were lower than in those who did not achieve the primary outcome, but was not statistically different (1.73 vs. 3.01 µg/mL, *P* = 0.32; Figure 2). No neutralizing antibodies were detected during the study period. Predictors of response or remission to intravenous UST reinduction were not identified in univariate or logistical regression analysis (age, sex, weight, smoking status, type of reinductions [IV or SC], concurrent use of immunomodulators, HBI pre-induction, CRP pre-induction, and pre-induction UST concentration).

### Safety

No serious adverse events were reported following UST reinduction. One patient experienced a minor infusion reaction of facial erythema and dyspnoea but was able to complete the dose at a lower infusion rate.

## DISCUSSION

Despite the increasing number of available therapies for patients with moderate to severe Crohn’s disease, there remains a subset of patients with refractory disease. In patients who have failed multiple biologics, it becomes imperative to optimize remaining therapies. To date, optimization strategies for biologic therapies have usually consisted of increasing the dose or shortening the dose interval. Very few data exist on reinduction strategies of any of the available biologic therapies. With respect to UST, there are limited retrospective data to support dose escalation during maintenance (15,16). Ollech et al. found that clinical remission in patients with an elevated HBI at baseline was achieved in 28% of patients who escalated to every 4-week dosing at 3 months. Kopylov et al found that clinical remission (defined by HBI) off corticosteroids was achieved in 18% of patients with dose escalation at week 16. Dose escalation in this study was mainly from every 8-week dosing to every 4-week dosing.

We present the largest cohort of patients undergoing UST reinduction. Our patients were relatively refractory, as 88% had already been dose optimised by decreasing the dosing interval to every 4 weeks at the time of inclusion. All patients included in this study had previously failed at least one anti-TNF agent, 74% had failed two biologics and 14% had failed three biologic therapies. Most patients had either a stricturing or penetrating phenotype and 40% had a history of perianal disease. Despite all these poor prognostic and clinical factors, nearly a third of patients achieved the primary end point of corticosteroid free clinical remission with either biochemical and/or endoscopic response or remission following UST IV reinduction. These rates are comparable to the subset of patients in the study of Kopylov et al. who also received IV reinduction. In that study, a similar endpoint of corticosteroid free clinical remission (among patients with an elevated HBI at baseline) was achieved 51% of those who received IV reinduction at a median follow-up time of 14 weeks. Baseline demographics differed between these studies and further comparisons of dose escalation to every 4 weeks and IV reinduction are needed to determine the optimal method of dose optimization. A recent study of 15 patients demonstrated that IV reinduction achieved clinical remission (by HBI) in 57% of patients (17). The previous largest study by Bermejo et al. included 53 patients with Crohn’s disease that received reinduction with UST due to a loss of response. At 16 weeks after reinduction, clinical remission (by HBI) was achieved in 43.3% whereas a clinical response was achieved in 52.8% (18). Another study by ten Bokkel Huinink et al. looked at drug survival in 31 patients with Crohn’s disease who received reinduction with UST. By weeks 20 and 53, 74% and 71% of patients were still on UST maintenance therapy. While the decision to continue UST was based on clinical, biochemical and endoscopic improvement, the ultimate decision was left at the discretion of the treating physician (19). Our study shows a comparable degree of clinical outcome to UST re-induction with 51.3% of patients achieving clinical remission and an additional 10.3% having a clinical response. The use of a composite primary outcome combining clinical remission with the more objective biochemical and/or endoscopic response adds an extra degree of robustness to highlight the benefit reinduction with IV UST. Another important consideration is whether the benefits of ustekinumab reinduction are dependent on initial induction strategies. In this study, of the 58 patients assessed at follow-up, 29 received initial induction with IV ustekinumab compared to 28 that were induced with SC ustekinumab. The findings were similar between groups with 27.6% (*n* = 8) of the IV subgroup achieving the primary outcome compared to 35.7% (*n* = 10) of the SC subgroup. These findings suggest that IV ustekinumab reinduction is beneficial to patients regardless of the modality they were initially induced with.

One of the interesting questions raised in this cohort is whether some patients require higher UST levels than previously considered therapeutic in order to respond. Prior studies have reported an association between serum UST levels and efficacy. In the IM-UNITI cohort, a trough level of ≥1 μg/mL (electrochemiluminescent assay, ECLIA) was associated with significantly higher rates of clinical remission (14). In a prospective study, Battat et al. reported that trough levels above 4.5 μg/mL (homogeneous mobility shift assay, HMSA) were associated with a decrease in biomarkers and with endoscopic response (20). This wide range is likely explained by the two different assays used in these studies. Verdon et al. recently demonstrated that the UST HMSA yields approximately three-fold higher absolute values than ELISA/ECLIA (21). In our population of refractory patients, we demonstrated a response to IV reinduction even in patients who already had therapeutic levels according to previously defined thresholds (89% of patients had baseline trough concentrations of >1 μg/mL). A “therapeutic” concentration of UST should therefore not preclude use of IV reinduction in the setting of partial response or LOR. Although the mean trough concentration pre-induction was numerically lower in patients achieving the primary outcome compared to those who did not, we could not identify a cut-off that was associated with non-response. Consistent with previously reported low rates of immunogenicity, we did not detect any neutralizing antibodies either pre- or post-reinduction. These findings warrant further studies to identify the optimal target trough concentration of UST.

This is the largest study to provide real world data on the therapeutic approach of IV UST reinduction in North American and European CD patients with partial response or LOR to maintenance UST therapy. The strengths are the use of robust outcomes according to standardized scoring systems. Our study does however have some limitations. For one, patients received IV UST reinduction at the discretion of their treating physician without predetermined definitions of incomplete response or loss of response. Given its retrospective nature, data collection was dependant on proper documentation and was limited by missing data. For instance, 11 of the 58 patients that were still on UST maintenance therapy at follow-up did not have an available HBI; therefore, clinical activity was based off the treating physician’s clinical judgement. Furthermore, subtle details such as abdominal mass or arthralgias may have been missed when calculating HBI for lack of a standardized scoring sheet. Otherwise, some of the outcome measures that were used for the composite primary endpoint were missing. For example, only a small number of patients had documented endoscopic assessment pre- or post-IV reinduction. However, given the substitutive nature of the components used for the composite primary outcome, any missing data is likely to result in a more conservative estimate as to whether response or remission was achieved. Furthermore, there was no central reading of endoscopies. Another limitation resulting from our retrospective data collection was the variable time to follow up which could affect the measured response rates. Finally, there was the possibility for selection bias, given only the patients with pre-induction data and then a follow up assessment were included in the analysis of outcomes.

In conclusion, even in the setting of refractory Crohn’s disease, ustekinumab IV reinduction is a well-tolerated and effective option to induce response and remission in patients with partial response or loss of response to maintenance UST therapy. Further prospective studies evaluating this strategy are warranted.
